# Concurrent validity and discriminative ability of Dutch performance-based motor tests in 5 to 6 years old children

**DOI:** 10.1371/journal.pone.0224722

**Published:** 2019-11-20

**Authors:** Marlou L. A. de Kroon, Willem G. van Kernebeek, Britta F. Neve, Jessica M. ter Veer, Sijmen A. Reijneveld, Henrica C. W. de Vet, Huub M. Toussaint

**Affiliations:** 1 Department of Health Sciences, University Medical Center Groningen, University of Groningen, Groningen, The Netherlands; 2 Faculty of Sports and Nutrition, Amsterdam University of Applied Sciences, Amsterdam, The Netherlands; 3 Department of Epidemiology and Biostatistics, Amsterdam Public Health Research Institute, VU University Medical Center, Amsterdam, The Netherlands; Universitat Konstanz, GERMANY

## Abstract

**Aim:**

To assess the concurrent validity and discriminative ability of total, gross and fine motor (TM, GM and FM) scores of Dutch performance-based motor tests, the Baecke-Fassaert Motor Test (BFMT) and the 8- and 4-Skills Scan (SkSc) with the Movement Assessment Battery (MABC) for children at age 5.

**Method:**

116 Dutch children (40.3% boys) were included. Spearman’s rho correlations and area under the curves (AUC) were assessed.

**Results:**

Correlations between the TM scores of the tests were strong (absolute values from 0.58 to .65); the correlations between the GM scores and the FM scores between and within tests were weaker (absolute values from 0.30 to 0.45). Related to the cut-off (15^th^ percentile) of the MABC, the AUC of the BFMT, 8- and 4-SkSc, the AUC was 0.853 (95% CI: 0.757–0.949), 0.905 (95% CI: 0.837–0.972) and 0.844 (95% CI: 0.730–0.957), respectively. At optimal cut-offs, the sensitivity and specificity of the BFMT, the 8- and 4-SkSc were 78.6 and 78.4%, 92.2 and 73.2%, 78.6 and 76.3%, respectively.

**Conclusion:**

All tests had a reasonably high discriminative ability, but validation with the MABC-2 and adaptations are needed to meet the requirements for screening (i.e. sensitivity ≥80% and specificity ≥90%). The relatively weak correlation between GM and FM scores implies that tests should be normalized and validated for GM and FM ability, separately.

## Introduction

A growing number of children has a motor delay as an isolated or more general developmental problem.[1] This may be partly due to increasing frequencies of preterm births resulting in a higher prevalence of congenital developmental problems, especially Developmental Coordination Disorder (DCD),[2] with a prevalence in childhood of around 5 to 15%.[3] Another reason may be the increasing frequency of under-stimulation due to the decreasing levels of physical activity in children.[4]

Finally, the increased awareness of the relevance of motor development among both care providers and parents may also have led to an increase in the identification of these problems. Children with motor developmental problems often feel less socially accepted, and have a lower self-efficacy and higher levels of anxiety.[5–7] Consequently, these children participate less in physical activities, increasing the risk of becoming overweight and hence further deterioration of physical and psychosocial health. The resulting cumulative effects of motor developmental problems and under-stimulation may lead to an even relatively bigger deviation from normal development at later ages.[8]

Early assessment could help to minimize the adverse consequences of DCD and other motor problems. The earliest age at which DCD usually is assessed is 5 years.[9] Assessment before age 5 is not recommended, unless there is a severe impairment assessed through at least two motor assessments carried out at least 3 months apart.[9,10] Around the age of 5 years, children’s basic motor skills should be sufficiently developed to be able to learn more advanced motor skills needed for physical fitness, and academic, social, and emotional functioning. Therefore, children with any other underlying cause of a motor developmental problem can benefit from an assessment at age 5. From this age onwards, stimulating learning environments and a pro-active approach of schools may prevent or reverse an ongoing accumulation of the effects of under-stimulation of children with a diagnosis as DCD, or of children who have a motor developmental problem due to under-stimulation.[9]

In the Netherlands, Preventive Child Health Care (PCHC) professionals and Physical Education (PE) teachers at primary schools assess the motor development at age 5, independently from each other. They most often choose for performance-based tests, as these are often assumed to give a more objective impression of children’s skills, which has also been described for the assessment of language development.[11] The PCHC professionals use the Baecke-Fassaert Motor Test (BFMT)[12–14] to assess gross and fine motor (GM and FM) skills, and a large number of the Dutch primary schools use the 8- or 4-SkSc [15–18] to assess GM skills at this age. On the basis of the test results, medical decision making is supported (e.g. a referral to a physiotherapist).

In contrast to validated questionnaires on motor skills, evidence lacks regarding the discriminative ability of these frequently applied Dutch performance-based tests. Therefore, our first aim was to investigate the concurrent validity and discriminative ability of the BFMT and the 8-SkSc and the less extensive version of this test, the 4-SkSc, with the Dutch version of the MABC, for 5-year-old children.

Typically, motor skills are categorized into two groups: gross motor versus fine motor skills, which represent different aspects of motor development. Delay in GM and FM skills require different types of treatment.[19,20] Therefore, the second aim was to assess the concordance between the total (TM), the GM and FM motor sub-scores within and between tests, because the 8- and 4-SkSc are used to test GM skills only. The MABC is used worldwide and is considered to be a reliable and valid instrument for assessing motor skills,[21] and has been used in numerous studies to assess the validity and discriminative ability of motor tests.[22–25]

## Methods

### Medical ethical statement

The study protocol has been approved by Ethics Committee Human Movement Sciences, VU University Amsterdam, and the Committee informed us that the rules laid down in the Medical Research Involving Human Subjects Act (also known by its Dutch abbreviation WMO), did not apply to this research proposal. Oral consent was obtained from the parents after informing them that data would be analyzed anonymously.

### Design, setting and population

In this cross-sectional study, in 2012–2014 we included 5-year-old children from three general primary schools in Amsterdam during their physical education lessons. We included children from schools situated in neighborhoods with a different socio-economic status (SES), aiming at a wide variety of motor skills as SES is highly related to motor skills.[26] In addition, the distribution of ethnicity among the children of these schools is fairly comparable to the distribution in all primary school children in Amsterdam.[27]

Inclusion criteria were the following: (1) the child had no medical history with problems, such as a physical or neurological disability (which is registered at these schools with permission of the parents), that could influence motor development, and (2) the child had an IQ rating ≥70 (children with an IQ below 70 are recognized by the teachers at an younger age and placed at a special education school; in case a placement has not yet been realized, it is known at school that the child has special education needs).

First, written consent was obtained from schools to participate in the study before parents of children were asked to participate in the study. Then, parents of all children and the teachers were informed during an information meeting and handed a written information letter, accompanied with the opportunity to object or opt-out.

### Sample size calculation

The power calculation was based on the binormal approximation to the standard errors of the AUC.[28] On the basis of the cut-off at the 15^th^ percentile of the MABC, the total sample size needed was N = 99, in order to find an AUC of 0.8 as significantly different from 0.6 (which we assumed that would be the AUC on the basis of medical judgment by professionals), with a power of 80% and a confidence of 95%.

### Procedure

All children were tested on the same day. The first child started with the MABC (followed by the BFMT and the 8- and 4-SkSc), the second with the BFMT (followed by the 8- and 4-SkSc and the MABC), and the third with the 8- and 4-SkSc (followed by the MABC and the BFMT), and so on. All motor tests were performed by two assistants, who were trained in applying and scoring the tests according to strict protocols.

### Measurements

#### Baecke Fassaart Motor Test (BFMT)

The BFMT assesses GM and FM skills for age 5 to 6.5 years, and takes about 10 minutes per child[13,14] (see Table 1). Until now, the BFMT has not been studied extensively. It has been normed for Dutch children aged 5 to 6,5 years (n = 1800), in primary education in 1984. In a research in 2016, the BFMT has been compared with the Dutch version of the MABC-2 (n = 61), showing a fair value of the Cohen’s kappa (0.45; 95% CI: 0.28–0.61).[29] The assessment of each item consists of two categories: sufficient (score = 1) and insufficient motor control (score = 0). The maximum score is 13 points. Finally, the total (or sum) scores of these items can be compared with age- and gender-specific scores of a norm group, which have been transformed into percentiles. In PCHC practice the cut-off score is set at the 10th percentile of the score of a norm group (P10), corrected for age and gender. A score below this cut-off is considered as insufficient.[13,14]

**Table 1:** The items of (the subscales of) the MABC, the BFMT and the 8- and 4-SkSc.

Subscales	MABC	BFMT	8- and 4-SkSc
**Fine motor skills**	• posting coins in a bank box	• copying figures	*Not applicable*
	• drawing a line into a trail	• drawing a line into a trail	
	• threading beads	• putting dots	
		• finger-thumb opposition	
		• eye movements	
		• top-nose test	
		• diadochokinesis	
		• tying shoelaces	
**Gross motor skills**			
Ball skills	• catching a bean bag	*Not applicable*	• bouncing ball *
	• rolling a ball into a goal		• catching a ball
Balance	• one-leg balance	• one-leg balance	• standing on one leg
	• jumping over a cord	• heel walking	• balancing (on a beam)*
	• walking heels raised on a line	• walking on a line	
Locomotion and others	*Not applicable*	• hopping	• jumping force *
		• jumping over a line	• jumping coordination*
			• climbing
			• rolling over

* These items belong to both the 8- & 4-SkSc; the other items only belong to the 8-SkSc

MABC Movement Assessment Battery for Children, BFMT Baecke Fassaart Motor Test, SkSc Skills Scan

### 8- and 4-Skills Scan (8- and 4-SkSc)

The 8- and 4-SkSc, both normalized for Dutch children, both assess GM skills for the ages 2 to 13 years. The 4-SkSc is the result of an experts’ consultation to make the 8-SkSc more feasible, resulting in a choice for four items which are most discriminating, but still covering the three domains of the 8-SkSc (see Table 1). The 4-SkSc has been proven a reliable test,[17] and includes the following items: bouncing (ball), one-leg balance, jumping force, and jumping coordination. Each sub-scale of the 8- and 4-SkSc is divided into 9 difficulty levels, matching a certain calendar age between age 2 and 13. Therefore, the 8- and 4-SkSc can be seen as a matrix with 8 (or 4) (sub-scales) times 9 possibilities, and Motor Age as the main outcome measure. For the 4-SkSc, raw data were converted into Motor Age as follows:

$$Motor\;Age=\frac{\left(level\;\prime balance\prime +level\;\prime jumping\;force\prime +level\;\prime jumping\;coordination\prime +level\;\prime bouncing\;ball\prime \right)}{4}$$

For the 8-SkSc, raw data were converted likewise, whereby the nominator equals 8 and the denominator equals the sum of the scores of all 8 sub-scales. Testing takes about 8 and 16 minutes per participant, respectively.

### MABC

The MABC, validated in the Dutch population, was used as reference standard, assessing both GM and FM skills. We used the first version of the MABC, because at the time of the measurements, the MABC-2 had not yet been fully implemented in the Netherlands. Furthermore, at that moment, most other motor screening tests have been validated with this version, enabling a better comparison between studies.

In the Netherlands, the MABC is mandatory to assess the diagnosis DCD.[30,31] See Table 1. Using the first version of the MABC results in a Total Impairment Score, implying the higher the score the lower the motor skills. The MABC takes about 30 minutes per participant. In assessing the sensitivity and specificity of the BFMT, and the 8- and 4-SkSc, we used the 15^th^ percentile cut-off of the total MABC score (i.e. 9.5), including both definite and borderline motor problems.[24,30] Both conditions need follow-up and/or treatment.[24] Since for the GM and FM scores of the MABC, no 15^th^ percentile cut-offs have been assessed, we also used the 15^th^ percentile cut-off of the TM, GM, and FM scores as assessed in our study population. These corresponded to a TM, GM and FM score of 9.0, 7.0 and 3.7, respectively.

### Classification of gross and fine motor skills

Typically, motor skills are categorized into two groups: gross motor skills versus fine motor skills. Gross motor skills are involved in movement and coordination of the arms, legs, and other large body parts, fine motor skills in smaller movements in wrists, hands, fingers, feet and toes. Together, they to provide coordination, whereby sometimes, the contribution of these separate skills are hard to distinguish. Therefore, we choose to classify the motor skills into these two categories as much as possible in line with the developers of the three tests.[14,15,21,31] The same principal was applied for the subdivision of gross motor skills into ball skills/object manipulation, balance, and locomotion. One exception was the top-nose test, which was not classified by the developer.[14] We classified this test item as part of the fine motor items as intention tremors which can be assessed by the top-nose test are related to the functioning of the fine distal motor functioning.[32]

### Statistical analyses

We assessed the concurrent validity between the TM, GM and FM scores within and between the tests, on the basis of Spearman’s correlation coefficients, as the data were not normally distributed. A correlation coefficient less than 0.3, was considered weak, between 0.3 and 0.5 moderate, and above 0.5 strong.[33] The discriminative ability of the BFMT and the 8- and 4-SkSc was assessed by calculating the area under the ROC-curve (AUC). For the reference values of the TM scores of the BFMT and the 8- and 4-SkSc we used the cut-off of the MABC, representing the 15^th^ percentile score in the reference population,[31] and the 15^th^ percentile based on our own study. However, the 15^th^ percentiles of the reference population are not assessed for the MABC GM and FM sub-scores, separately. Therefore, for the GM and FM scores of the tests the cut-off related to the 15^th^ percentile of the MABC was used on the basis of our own study population, only.

Sensitivity and specificity of the motor scores were calculated at optimal cut-offs using the MABC as the reference standard. Optimal cut-off values were calculated with the Youden Index.[34] For the BFMT, also sensitivity and specificity were calculated at the currently applied cut-off point (which is not necessarily equal to the optimal cut-off, as calculated in this study). The usual requirement for screening tests is a sensitivity of at least 80% and a specificity of at least 90%.[35] A pairwise handling of missing data was used. There were missing values in one of the test-items of the 8- or 4-SkSc of 5 children. Therefore, the analyses were based on n = 111 (involving the 8- or 4-SkSc) or n = 116 (not involving the 8- or 4-SkSc). Analyses were performed with SPSS statistical software, version 21.0 for Windows (SPSS Inc. Chicago ILL).

## Results

### Background characteristics

In total 116 children, 48 boys (40%) and 71 girls (60%), participated in the study. Their mean age was 5.6 years (SD 0.28). In total 14 children fell below the 15^th^ percentile, according to the MABC-1. For 5 children, the tests results of the 8- and 4-SkSc were missing, because of missing test items. The gender and age distribution of the schools, which were situated in neighbourhoods with a different socio-economic status, is shown in Table 2.

**Table 2:** Background characteristics and scores of the motor tests of the children (N = 116) in relationship to the characteristics of the schools.

	School: number (socioeconomic status scores in z-scores*, [27,36] non-western ethnicity in %)			
	1 (0.30; 48.4%)	2 (1.15; 10.1%)	3 (-1.87; 36.0%)	Total
*Number of children*	30	22	64	116
boys (%)	16 (53.3%)	9 (40.9%)	23 (35.9%)	47 (40.3%)
girls (%)	14 (46.7%)	13 (59.1%)	41 (64.1%)	69 (59.7%)
Mean age in years (SD)	5.51 (0.31)	5.36 (0.31)	5.52 (0.29)	5.55 (0.28)
*Scores of the tests (mean (SD); median (min*, *max)*				
MABC TM	3.40 (3.45) 2.75 (0.00, 13.00)	5.36 (4.97) 5.00 (0.00, 16.00)	3.88 (4.24) 2.25 (0.00, 17.00)	4.04 (4.22) 3.00 (0.00, 17.00)
MABC GM	2.42 (2.72) 1.25 (0.00, 9.00;)	3.30 (3.16) 2.50 (0.00, 10.00)	2.85 (3.37) 2.00 (0.00, 14.50)	2.82 (3.16) 2.00 (0.00, 14.50)
MABC FM	0.83 (1.50) 0.00 (0.00, 5.50)	2.07 (2.18) 1.75 (0.00, 6.50)	1.03 (1.61) 0.00 (0.00, 6.50)	1.18 (1.75) 0.00 (0.00, 6.50)
BFMT TM	9.33 (1.86) 9.00 (5.00, 13.00)	8.55 (2.30) 8.5 (5.00, 13.00)	8.94 (2.27) 10.00 (4.00, 13.00)	8.97 (2.18) 9.00 (4.00,13.00)
BFMT GM	4.30 (0.88) 5.00 (2.00, 5.00)	4.18 (1.01) 5.00 (2.00, 5.00)	4.22 (0.95) 4.50 (2.00, 5.00)	4.23 (0.94) 5.00 (2.00, 5.00)
BFMT FM	5.03 (1.56) 5.00 (2.00, 8.00)	4.36 (1.87) 4.00 (1.00, 8.00)	4.21 (0.95) 5.00 (2.00, 8.00)	4.73 (1.67) 5.00 (1.00, 8.00)
8-SkSc	5.17 (0.28) 5.13 (4.75, 6.13)	5.04 (0.46) 5.00 (3.75–5.88)	5.23 (0.45) 5.25 (3.63–6.25)	5.18 (0.42) 5.13 (3.63, 6.25)
4-SkSc	5.28 (0.42) 5.25 (4.50, 6.75)	5.14 (0.56) 5.00 (4.00, 6.25)	5.43 (0.58) 5.25 (3.75, 7.25)	5.33 (0.54) 5.25 (3.75, 7.25)

MABC: Movement Assessment Battery for Children; BFMT: Baecke Fassaart Motor Test, SkSc: Skills Scan

* The socioeconomic status scores (SES), which are made available by the Social and Cultural Planning Office (SCP) in The Hague, the Netherlands, are composed of three elements: income, employment and education level. This status scores can be considered as a global estimation of the socioeconomic status of the parents of the children at these schools.[36]

### Concurrent validity

Table 3 lists the correlations between the TM, GM and FM scores of the MABC, BFMT, and the 8- and 4-SkSc for all children. All correlations were significant (p<0.01). The correlations of the TM scores of the Dutch motor tests, the BFMT, the 8- and 4-SkSc, with the MABC were strong: *r* = -.58, -.65 and -.56, respectively. The absolute values of the correlations between GM subscores and FM subscores, within or between tests, were much lower. The correlation of the GM scores of the three Dutch motor tests with the MABC GM and the correlation of the FM scores of the BFMT with the MABC FM were quite different. The correlation between the gross motor scores of the BFMT and 8-SkSc with the MABC GM was strong, whereas the correlations of the 4-SkSc with the MABC GM and the FM of the BFMT and the MABC FM were moderate (see Table 3). The correlation between the total score and the sub-scores within tests also differed, remarkably. The relationship of the BFMT total and the BFMT FM was much stronger than the correlation between the scores of the BFMT total and BFMT GM, whereas a stronger correlation was found for the MABC total and the MABC GM than for the MABC total and the MABC FM.

**Table 3:** Spearman’s correlation coefficients (with 95% CI) between the MABC, the BFMT and the 8- and 4-SkSc (N = 111).

	MABC	MABC	BFMT	BFMT	BFMT	8-SkSc	4-SkSc
	GM	FM	TM	GM	FM	GM	GM
MABC TM	0.93 (.90; .95)	0.68 (.55; .77)	-.58 (-.69; -43)	-.63 (-.75; -.50)	-.40 (-.56; -.22)	-.65 (-.76; -.53)	-.56 (-.68; -.42)
MABC GM		.41 (.24; .56)	-.53 (-.64; .37)	-.65 (-.76; -.52)	-.34 (-.50; -.15)	-.63 (-.73; -.49)	-.51 (-.64; -.35)
MABC FM			-.48 (-.61; -.33)	-.39 (-.54; -.21)	-.40 (-.55; -.23)	-.45 (-.59; -.28)	-.43 (-.57; -.26)
BFMT TM				.68 (.56; .78)	.92 (.88; .94)	.48 (.31; .61)	.44 (.27; .57)
BFMT GM					.36 (.19;.52)	.58 (.43; .70)	.49 (.33; .63)
BFMT FM						.32 (.14 to .48)	.30 (.11; .45)
8-SkSc GM							.83 (.75; .89)

MABC Movement Assessment Battery for Children, BFMT Baecke Fassaart Motor Test, Skills Scan SkSc

### Discriminative ability

Table 4A displays the AUC and the sensitivity and specificity for the BFMT and 8- and 4-SkSc related to the cut-off of the MABC, representing the 15^th^ percentile score in the reference population.[24] These ranged from 0.844 to 0.905. The sensitivity and specificity of these tests at optimal cut-offs ranged from 78.6 to 92.2% and from 73.2 to 78.4% respectively. It should be noted that the sensitivity and specificity of the BFMT at currently applied cut-offs (P10) were quite different, and especially the sensitivity was very low (i.e. 35.7%).

**Table 4:** AUC, sensitivity, specificity at optimal cut-off values of the BFMT, the 8- and 4-SkSc, using the total motor (TM) scores using the MABC as reference, with the 15^th^ percentile in the reference population (a)[31], and using the TM, gross motor (GM) and fine motor (FM) scores, using the MABC as reference, with the 15^th^ percentile of the study population (b).

***a*. *MABC (15***^***th***^ ***percentile) as reference***			
***Total Motor Scores***	BFMT*	**8-SkSc**	**4-SkSc**
N	116	111	111
Cut-off score	7.5	5.1	5.1
Sensitivity (%)	78.6	92.2	78.6
Specificity (%)	78.4	73.2	76.3
AUC (95%CI)	0.853 (0.757–0.949)	0.905 (0.837–0.972)	0.844 (0.730–0.957)
***b*. *Study population (15***^***th***^ ***percentile) as reference***			
***Total Motor Scores***	**BFMT**	**8-SkSc**	**4-SkSc**
N	116	111	111
Cut-off score	7.5	5.1	5.1
Sensitivity (%)	77.8	94.4	72.2
Specificity (%)	80,6	76.3	77.4
AUC (95%CI)	0.869 (0.786–0.952)	0.903 (0.843–0.963)	0.828 (0.726–0.929)
***Gross Motor*** ***Scores***	BFMT*	**8-SkSc**	**4-SkSc**
N	116	111	111
Cut-off score	4.5	5.1	4.9
Sensitivity (%)	100	88.2	47.1
Specificity (%)	61.2	74.5	92.6
AUC (95%CI)	0.884 (0.818–0.949)	0.870 (0.790–0.951)	0.746 (0.613–0.878)
***Fine Motor*** ***Scores***	**BFMT**	**8-SkSc**	**4-SkSc**
N	116	NA	NA
Cut-off score	4.5	NA	NA
Sensitivity (%)	68.8	NA	NA
Specificity (%)	64.0	NA	NA
AUC (95%CI)	0.709 (0.585–0.834)	NA	NA

* At the currently applied cut-off score, the sensitivity = 35.7 and the specificity = 93.1%. MABC Movement Assessment Battery for Children, BFMT Baecke Fassaert Motor Test, Skills Scan SkSc, AUC area under the curve, CI confidence interval, NA Not applicable

**Fig 1:**
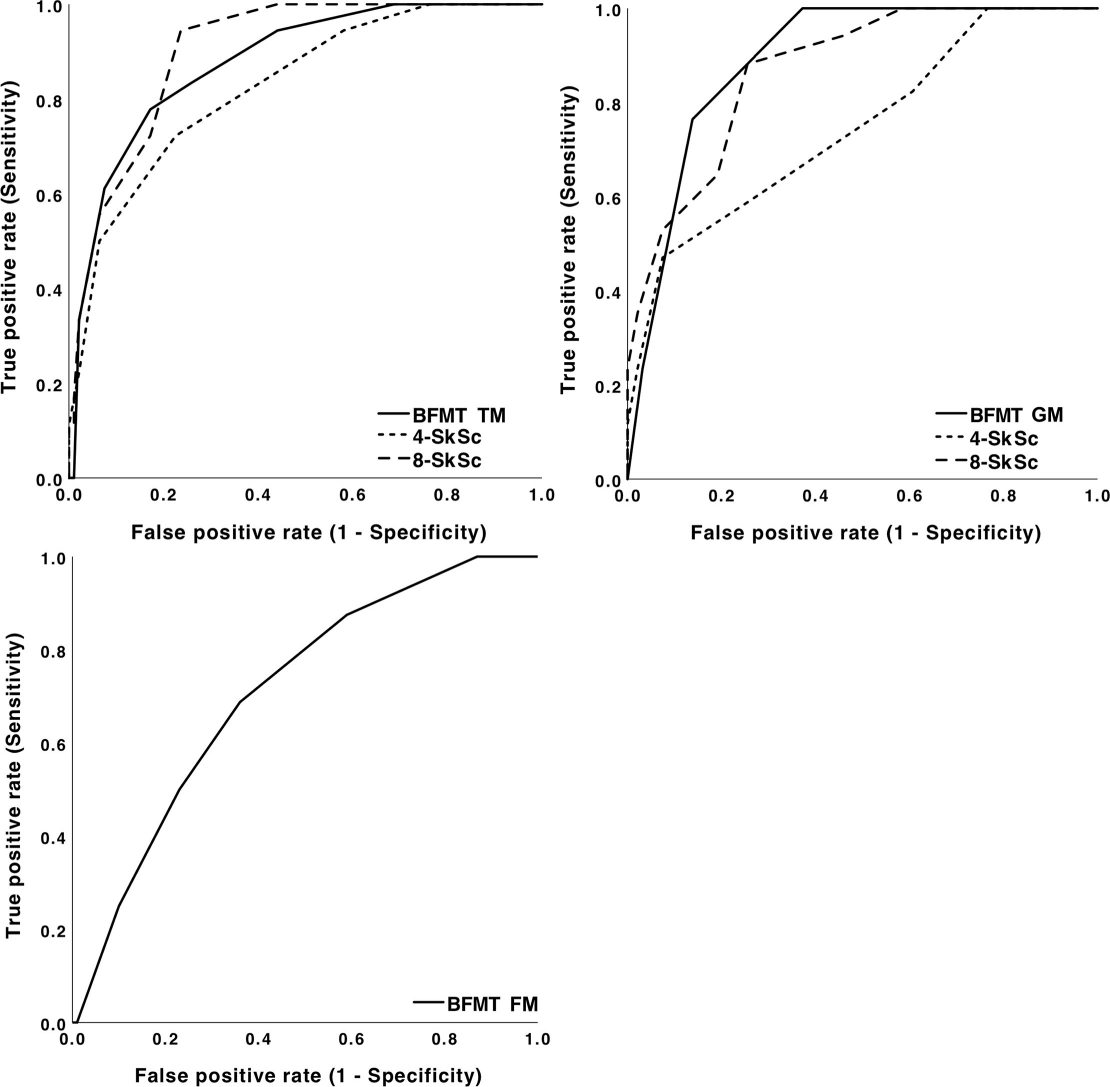
ROC plots of the BFMT (N = 116) and the 8- and 4-SkSc (N = 111) for the total scores (Fig 1A), the gross motor scores (Fig 1B), and the fine motor scores (Fig 1C) of the BFMT, related to the cut-off values of the total, gross and fine motor scores respectively (i.e. the 15^th^ percentile) of the MABC, assessed in this population of 5 years old children.

Table 4B displays the results related to the 15^th^ percentile scores calculated on the basis of our own study population for TM, GM, and FM scores. Fig 1 displays ROC-curves for all analyses. For the TM scores, the results were very similar to the results when using the 15^th^ percentile of the MABC in the reference population. The AUC’s for the GM scores of the BFMT and the 8- and 4-SkSc using the 15^th^ percentile of the study population ranged from 0.746 to 0.884. With respect to the GM scores, the sensitivity was highest for the 4-SkSc and the specificity was highest for the BFMT. The AUC for the FM score of the BFMT was 0.709, with a sensitivity of 64.0 and a specificity of 68.7% at optimal cut-offs. Based on the information in Table 4, the numbers of children that have been categorized correctly as having motor problems or having no motor problems on basis of the three motor skill tests can be found in Table 5.

**Table 5:** Children with a motor developmental problem according to the MABC-1 in relationship to an insufficient and sufficient score on the screening tests.

Screening test	Score	Motor developmental problem according to MABC-1	
		yes	No
**BFMT (optimal cut-off)**	Insufficient	11	22
*(n = 116)*	Sufficient	3	80
**BFMT (original cut-off)**	Insufficient	5	7
*(n = 116)*	Sufficient	9	95
**8-SkSc**	Insufficient	13	26
*(n = 111)*	Sufficient	1	71
**4-SkSc**	Insufficient	11	23
*(n = 111)*	Sufficient	3	74

MABC: Movement Assessment Battery for Children; BFMT: Baecke Fassaart Motor Test; Skills Scan SkSc

## Discussion

Our aim was to investigate concurrent validity and discriminative ability of frequently applied, performance-based, Dutch motor tests, the BFMT and the 8- and 4-SkSc with the MABC as reference standard, in the general Dutch population, for the total, GM and FM scores. Optimal cut-offs of the 8 and 4-SkSc and the BFMT were assessed. For the BFMT the sensitivity and specificity were calculated for both the optimal and currently used cut-offs.

### Concurrent validity and discriminative ability

We found strong correlations between the TM and GM scores of the three Dutch motor tests (i.e. the BFMT and the 8- and 4-SkSc) and the MABC. Our results with respect to the 4-SkSc are in line with other study results of our group in which we found exactly the same correlation of the 4-SkSk with the MABC-2 for 5–9 years olds as in this study with the MABC for 5-year olds (i.e. 0.56).[18] On beforehand, we would have expected differences at higher age-bands, since the MABC-2 contains essentially other test items at these age-bands.

The correlation between the BFMT FM and the MABC FM was moderate. The correlations between the GM and FM scores within the four tests and between the four tests were low to moderate, indicating that GM and FM skills should be considered as separate aspects of motor skills. At optimal cut-offs–as determined by the Youden Index–the discriminative ability of the TM scores of the BFMT and the 8- and 4-SkSc was reasonably high. The discriminative ability of the GM scores of these three tests was moderate to high, whereas the discriminative ability of the FM score of the BFMT was moderate. It should be noted that the sensitivity of the BFMT, with the use of currently applied cut-offs was very low. This may be caused by the fact that currently applied cut-offs have not been based on ROC analysis and generally, in practice, a higher specificity is more valued by preventive health care professionals. Moreover, the currently applied cut-off value, which is the 10^th^ percentile (P10), does not match the cut-off value of the MABC, which–in case of the MABC–is the 15^th^ percentile.[21,31]

The sensitivity and specificity at optimal cut-off values of none of the scores of the BFMT and the 8- and 4-SkSc met the criteria for diagnostic accuracy for screening of motor developmental problems (i.e. ≥80% and ≥90%, respectively.[35] However, the correlations and diagnostic accuracy of the TM and GM scores were close to acceptable These unfavorable findings of concurrent validity and diagnostic accuracy are in line with other studies on performance-based motor skill tests, with exception of a study about the MOT 4–6, which had a high sensitivity (88%) and specificity (90%) in a population of Belgian children.[37] However, in comparison with the Dutch motor tests, the MOT 4–6 takes some more time, i.e. 15 to 20 minutes, in comparison to 10, 16 and 8 minutes for the BFMT, the 8- and 4-SkSc, respectively.

Remarkably, at optimal cut-offs of the BFMT GM, the sensitivity was 100%, whereas the specificity was only 61.2%. For the next most favourable cut-off, according to the Youden index, the sensitivity was 72.2 and the specificity 86.7%. This phenomenon may be explained by the fact that the scales of the sub-items of the BFMT are dichotomous, whereas the sub-items of especially the 8- and 4-SkSc have more categories, allowing for a more precise assessment of motor skills development, especially by the 8-SkSc.

In studying the correlations between the sub-scores and the TM scores of the tests, we found a stronger correlation between the MABC TM and the MABC GM, than between the MABC TM and the MABC FM. This is in line with the study of Van Waelvelde et al.[22,23] In contrast, we found a stronger correlation between the BFMT TM and the BFMT FM, than between the BFMT TM and the BFMT GM. These findings are possibly due to the fact that 8 of the 13 items of the BFMT are fine motor skills, whereas in the MABC 5 of 8 subitems are gross motor skills. Thus, differences between the share of FM or GM test items might engender these outcomes. In addition, differences in motor construct between these tests might affect their mutual correlation.

The low correlations between GM and FM scores in general and the large differences between proportions of GM and FM test items of the MABC, BFMT, and the 8- and 4-SkSc, may partly explain why the BFMT and the 8- and 4-SkSc do not meet the requirements for screening yet. This may possibly negatively influence the validity of the tests, especially since it also has been shown that GM and FM skills represent different aspects of motor ability.[22,23] These results underpin the statement made in the study of Fransen et al.,[38] that different motor tests should be used for different aims.

### Strengths and limitations

An important strength of the study regards the use of designated test assistants, not having had previous contacts with these children, and the application of all tests on the same day whereby the first test for the children differed randomly, preventing sequence effects. Another strength is that we studied the motor tests in a general population of healthy children from primary schools. A limitation may be that the MABC may not be a perfect reference test, because it has been argued that some skills, such as handwriting, and functional performance are not being measured by the MABC (1 or 2).[39] However, the MABC currently is considered to be the best choice as reference test.

Although we used the previous (generally considered as outdated) version of the MABC, namely the MABC-1, we expect this to have little effect on the results of this study. The age-band for 5-year-old children of the MABC-2 is highly comparable to the age band of the first version of the MABC. For this age band, the basic skills that are being tested are the same for MABC-1 and MABC-2, and only two testitems have been replaced by comparable testitems. The test item “Rolling ball into goal’ has been replaced by “Throwing Beanbag onto Mat” which both assess throwing skills, and the test item “Jumping Over Cord” by “Jumping on Mats”, which both assess balance.[40] In addition, the correlations of a well-known motor test, the KTK, with the MABC-1 and the MABC-2 are equal,[41,42] implying that the differences between the MABC-1 and the MABC-2 are probably small. Although it could be argued that similar correlations do not imply that the sensitivity and specificity are similar as well, it is very likely that this is the case as the cut-off values and the test-items at this age band of the MABC-1 and MABC-2 are highly comparable.

Finally, the norm population of the MABC-1 (and MABC-2) is likely to be different with respect to ethnicity, although this has not been described for the MABC-1 or MABC-2.[31,39,41] This might have influenced the prevalence of motor problems in our population, as e.g. children from African or Indian descent are less prone to motor developmental problems. However, at least half of the variance in motor skills is likely to be influenced by the environment.[43] In addition, it is not very likely that the correlation between tests is influenced by ethnicity of the children.

It has been shown that the standard scores of the MABC-2 better meet, although not completely, the assumptions of normal distribution.[40,44] Therefore, we applied non-parametric tests to assess correlations of the tests with the MABC. Finally, with respect to the classification of sub-items of the BFMT in the two distinct categories fine and gross motor skills, we could have used an alternative classification by excluding the top-nose-test in the fine motor skills. This might have influenced the relationships between the different FM and GM sub-scores, but a post-hoc analysis showed that excluding the top-nose test from the FM skills slightly changed the results (AUC = 0.719 (95% CI: 0.593–0.846) instead of 0.709 (95% CI: 0.585–0.834).

### Implications

Before definitive conclusions can be drawn about the value of the BFMT, 8- and 4-SkSc as screening instruments, our study results need to be validated in other populations with the MABC-2. Subsequently, adaptations of the tests that better align the construct of motor skills with the MABC are recommended in order to improve the sensitivity and specificity. With respect to the BFMT, a relatively quick and easy to implement improvement would be to change the currently applied cut-offs of the BFMT to the optimal cut-offs. Notably, replacing the dichotomous scales of the sub-items of the BFMT by a scale with more categories or a continuous scale will probably also result in a better balance between sensitivity and specificity.

Another option, for which more efforts are needed, may be to assess which smallest number, least time consuming and practicable combination of motor test items from these tests and possibly other motor tests, such as the MOT 4–6,^33^ are most discriminative to assess motor development problems in the general population at this age. In addition, a stepwise approach in screening for motor developmental problems, by applying a sequential application of motor tests, may have added value for the cost-effectiveness in medical decision-making. Assessing cut-offs for GM and FM ability, separately, should be considered, as the correlation between GM and FM motor scores are low to moderate and therapeutic consequences of GM and FM problems differ. Finally, the decision on cut-offs should also be determined by whether a higher value is attached to a high sensitivity or, on the contrary, to a high specificity.

Although the studied Dutch tests do not meet the criteria for diagnostic accuracy for screening of motor developmental problems in community-based settings, we recommend continuing screening for motor delay at age 5 because of the possible serious academic and social impact of a motor.[5–7]. For this purpose, these three current Dutch motor screening tests could be applied, albeit it with caution. Hereto, extra anamnestic information could support medical decision making.
